# Clear distinction between *Burkholderia mallei* and *Burkholderia pseudomallei* using fluorescent *mot*B primers

**DOI:** 10.1186/s13028-015-0104-4

**Published:** 2015-03-07

**Authors:** Gernot Schmoock, Mandy Elschner, Lisa D Sprague

**Affiliations:** Friedrich-Loeffler-Institut, Bundesforschungsinstitut für Tiergesundheit, Naumburger Str. 96a, DE-07743 Jena, Germany; National and OIE Reference Laboratory for Glanders, Friedrich-Loeffler-Institut, Bundesforschungsinstitut für Tiergesundheit, Naumburger Str. 96a, DE-07743 Jena, Germany

**Keywords:** Duplex PCR, Fluorescent primers, *Burkholderia*

## Abstract

**Background:**

A frame-shift mutation in the flagellum motor gene *mot*B coding for the chemotaxis MotB protein of *Burkholderia mallei* has been utilized to design a conventional duplex PCR assay with fluorescent labelled primers.

**Findings:**

Species specificity was tested with a panel of 13 *Burkholderia* type strains. A total of 41 *B. mallei* field strains, 36 *B. pseudomallei* field strains, and 1 *B. thailandensis* field strain from different geographic regions were tested and correctly identified. Testing of 55 non-*Burkholderia* bacterial species revealed 100% specificity of the assay. The minimum detection limit was 1 pg DNA or 160 GE for *B. mallei* and 130 GE for *B. pseudomallei*, respectively.

**Conclusions:**

This assay enables the clear distinction between *B. mallei* and *B. pseudomallei*/*B. thailandensis.*

**Electronic supplementary material:**

The online version of this article (doi:10.1186/s13028-015-0104-4) contains supplementary material, which is available to authorized users.

## Findings

Despite *Burkholderia mallei*, *B. pseudomallei* and *B. thailandensis* being genetically closely related Gram negative bacteria, they display significant differences in pathogenicity and habitat. *B. mallei*, a facultative intracellular, non-motile, equine pathogen, is the causative agent of glanders, a highly contagious and frequently fatal zoonotic disease of the upper respiratory tract and lungs [1]. The disease has a 95% case fatality rate in untreated humans with septicaemia and a 50% case fatality rate in antibiotic treated individuals [1].

*B. pseudomallei*, a facultative intracellular, motile bacterium found in contaminated water and soil, is the etiological agent of melioidosis, an infectious disease in man and animal in the tropics [2]. The clinical picture in animals and humans resembles that of glanders in horses. Human infection usually develops after inhalation, ingestion, or cutaneous uptake of the pathogen [2,3]. Melioidosis has a case fatality rate of 39.5%, and untreated septicaemia is fatal in up to 80% of cases [4]. Both *B. mallei* and *B. pseudomallei* are considered potential bioweapons and are listed as category B biothreat agents by the U.S. Centers for Disease Control and Prevention [5]. *B. thailandensis* is generally considered a weakly pathogenic, motile soil bacterium, rarely causing disease in man or animal [6]. Glanders and melioidosis may cause diagnostic problems in endemic regions because of their clinical, morphologic and genetic similarity, and even more so in non-endemic countries, due to the lack of awareness of these diseases. In order to initiate appropriate patient treatment, rapid species identification is necessary, especially in view of the intrinsic resistance of both agents to many commonly used antibiotics and their differing susceptibilities [7,8].

Based on the results from a previous study [9], a frame-shift mutation in the flagellum motor gene *mot*B coding for the chemotaxis MotB protein [GenBank:BMA2861] of *B. mallei* (ATCC 23344) was utilized to design a simple conventional duplex PCR assay with fluorescent labelled primers enabling the distinction between *B. mallei* and *B. pseudomallei*/*B. thailandensis.* Bacterial strains were obtained from the strain collection of the National and OIE Reference Laboratory for Glanders at the Friedrich-Loeffler-Institute in Jena, Germany (Tables 1 and 2). All *Burkholderia* strains were cultured at 37°C on calf blood agar containing 3% (v/v) glycerol. All other bacteria were grown on standard media and appropriate atmospheric conditions.

**Table 1:** **Panel of** ***Burkholderia mallei*** **and** ***B. pseudomallei*** **field strains used for validation**

**Origin**	***B. mallei***	***B. pseudomallei***
**Africa**	-	2
**Arabian Peninsula**	3	-
**Asia**	-	1
**East Asia**	1	4
**South Asia**	17	5
**Southeast Asia**	-	13
**Europe**	3	4
**Indonesia**	1	-
**South America**	5	2
**Transcontinental Europe/Asia**	2	-
Unknown	9	5
**Total**	**41**	**36**

**Table 2:** **Panel of non-Burkholderia strains used for specificity testing**

**Species**	**Strain**	**Species**	**Strain**
*Actinobacillus pleuropneumoniae*	ATCC 27088	*Legionella pneumophila sub. pneumophila*	DSM 7513
*Bacillus atrophaeus*	ATCC 9372	*Mannheimia haemolytica*	ATCC 33396
*Bacillus brevis*	ATCC 8246	*Ochrobactrum anthropi*	CCUG 1047
*Bacillus cereus*	ATCC 10876	*Oligella urethralis*	DSM 7531
*Bacillus megaterium*	DSM 90	*Pasteurella multo ssp.multo*	ATCC 43137
*Bacillus mycoides*	ATCC 6462	*Pasteurella multocida*	DSM 5281
*Bacillus subtilis*	ATCC 6633	*Proteus mirabilis*	DSM 4479
*Bacillus thuringiensis*	ATCC 10792	*Pseudomonas aeruginosa*	ATCC 9027
*Bartonella henselae*	DSM 28221	*Pseudomonas alcaligenes*	ATCC 14909
*Bartonella quintana*	DSM 21441	*Pseudomonas fluorescens*	ATCC 13525
*Bordetella bronchiseptica*	ATCC 19395	*Pseudomonas polymyxa*	ATCC 842
*Brucella abortus*	ATCC 23448	*Pseudomonas putida*	ATCC 12633
*Brucella melitensis*	ATCC 23456	*Rhodococcus equi*	DSM 20307
*Brucella suis*	ATCC 23444	*Salmonella enteritidis*	147 (95)
*Campylobacter coli*	DSM 4689	*Salmonella typhumirium*	9098 (221)
*Campylobacter jejuni subsp. jejuni*	DSM 4688	*Staphylococcus aureus subsp. aureus*	DSM 6732
*Chlamydia abortus*	07 DC0059	*Stenotrophomonas maltophilia*	ATCC 13637
*Chlamydia pecorum*	06 DC0055	*Streptococcus agalactiae*	DSM 6784
*Chlamydia psittaci*	C1/97	*Streptococcus equi subsp. equi*	ATCC 9528
*Clostridium baratii*	ATCC 25782	*Streptococcus equi subsp. zooepidemicus*	ATCC 700400
*Clostridium botulinum A*	NCTC 7272	*Streptococcus equinus*	DSM 20558
*Clostridium botulinum B*	NCTC 7273	*Streptococcus parauberis*	DSM 6631
*Escherichia coli*	DSM 30083	*Taylorella equigenitalis*	DSM 10668
*Francisella tularensis sub. holarctica*	LVS	*Yersinia enterocolitica subsp. enterocolitica*	ATCC 9610
*Francisella tularensis sub. tularensis*	FSC 237 (SchuS4)	*Yersinia enterocolitica subsp. enterocolitica*	DSM 9499
*Haemophilus influenzae*	ATCC 9006	*Yersinia enterocolitica subsp. palearctica*	DSM 13030
*Klebsiella pneumoniae subsp. pneumoniae*	DSM 30104	*Yersinia pseudotuberculosis*	IP32953
*Lactobacillus ruminis*	DSM 20403		

Genomic DNA was prepared from culture material using the High Pure PCR Template Preparation Kit according to the manufacturer’s instructions (Roche, Mannheim, Germany). All DNA samples were quantified using a NanoDrop 1000 spectrophotometer (Fisher Scientific, Schwerte, Germany). The duplex polymerase chain reaction (PCR) was designed using the forward primer MBF04 (5′- CGTCAAGCGGGTGAACCA -3′), the 6-FAM labelled reverse primer MBR04-FAM (5′-6-FAM-GTCGTCCTCGCTCTTTCGC -3′), and the ATTO565 labelled reverse primer MBR10-ATTO565 (5′-ATTO565-GTCCTCGCTCTTCTTCGCG-3′). Primers were designed with the Genious software package (Ver. 6.1), to generate a specific 6-FAM labelled 326 bp DNA fragment for *B. mallei* and an ATTO565 labelled 325 bp DNA fragment for *B. pseudomallei*/*B. thailandensis,* respectively. Labelled primers were obtained from Microsynth (Balgach, Switzerland), the unlabelled primer from Jena Bioscience (Jena, Germany). PCR was conducted in a 20 μL reaction containing 0.3 μM of the primers (MBF04, MBR04-FAM, and MBR10-ATTO565), 1 × 5-Prime HotMasterMix (VWR, Darmstadt, Germany), 2.5% DMSO and 10 ng template (total DNA). The PCR was performed in a Mastercycler pro S™ (Eppendorf, Germany) under the following conditions: initial denaturation at 95°C for 1 min; 40 cycles at 95°C for 10 s, 63°C for 15 s, 70°C for 30 s, and the final extension at 70°C for 5 min. 13.3 μL PCR reaction mixed with 2.7 μL 6 × Loading Dye (Fermentas, Schwerte, Germany) were analysed by electrophoresis on a 1.25% agarose gel (wt/vol) at 9 V/cm for 40 min. Images were captured after an exposure period of 30 s for each LED/filter set using the G-Box EF2 Gel Documentation System (Syngene Europe, Cambridge, UK): Blue-LED/Filt525 and Green-LED/Filt605 for the visualisation of 6-FAM and ATTO565 labelled PCR products, respectively. For optional ethidium bromide imaging (302 nm UV illuminator/FiltUV), the gel was stained after capturing the 6-FAM/ATTO565 images. Fragment sizes (326/327 bp) and correct labelling (6-FAM/ATTO565) of the amplicons were confirmed by means of capillary electrophoresis using a Genetic Analyzer 3130 with a G5 filter set (Applied Biosystems/Hitachi, Darmstadt, Germany). Species specificity was tested with a panel of 13 *Burkholderia* type strains. Additionally, a total of 41 *B. mallei* field strains from equines, 36 *B. pseudomallei* field strains from human and environmental origin, and one *B. thailandensis* field strain, all from different geographic regions were tested and correctly identified (Table 1). Testing of 55 non-*Burkholderia* bacterial species revealed 100% specificity of the assay (Table 2). The minimum detection limit was 1 pg DNA or 160 genome equivalents (GE) for *B. mallei* and 130 GE for *B. pseudomallei*, respectively. In order to compare the sensitivity of our assay with other assays used by the National and OIE Reference Laboratory for Glanders, several clinical *B. mallei* samples were tested by a conventional *fli*P PCR [10] and a real time PCR assay targeting *fli*C [11]. Despite the lower sensitivity we determined for our assay, it revealed comparable sensitivity to the conventional *fli*P PCR and a higher sensitivity than the real time *fli*C assay in the tested clinical samples (Additional file 1).

Fluorescent primers are widely used in real time PCR technology and several highly sophisticated and elegant PCR assays have been developed for the identification and differentiation of *B. mallei* and *B. pseudomallei* and other *Burkholderia* species in the past few years [12]. This study describes the design of a simple conventional duplex PCR with fluorescent labelled primers for amplifying species-specific amplicons of *B. mallei* and *B. pseudomallei*/*B. thailandensis*, respectively. These closely related species can cause considerable problems during the identification process in the laboratory as colony characteristics and routine biochemical tests are not sufficiently discriminative for species identification. The benefit of this assay is not only the unambiguous identification of *B. mallei* and the closely related species *B. pseudomallei* and *B. thailandensis* by fluorescence image capturing but also the possibility of detecting the *B. mallei/pseudomallei*/*thailandensis* complex on a standard ethidium bromide stained agarose gel.

## Additional file

Additional file 1:
**Comparison of the** ***mot*** **B PCR assay to the conventional** ***fli*** **P and real time** ***fli*** **C PCR assays in clinical samples (** ***Burkholderia*** **type strains ATCC 23343 T, ATCC 23344T).**

## Abbreviations

ATCC: American type culture collection
CCUG: Culture collection university of Göteborg
DSM: Deutsche Sammlung von Mikroorganismen
FAM: Fluorescein
FSC: *Francisella* strain collection, Sweden
GE: Genome equivalent
LED: Light-emitting diode
NCTC: National collection of type cultures

## References

[1] Dvorak GD, Spickler AR (2008). Glanders. J Am Vet Med Assoc.

[2] Cheng AC, Dance DA, Currie BJ (2005). Bioterrorism Glanders and Melioidosis. Euro Surveill.

[3] Wiersinga WJ, Currie BJ, Peacock SJ (2012). Melioidosis. N Engl J Med.

[4] Zysk G, Splettstösser WD, Neubauer H (2000). A review on melioidosis with special respect on molecular and immunological diagnostic techniques. Clin Lab.

[5] Rotz LD, Khan AS, Lillibridge SR, Ostroff SM, Hughes JM (2002). Public health assessment of potential biological terrorism agents. Emerg Infect Dis.

[6] Brett PJ, DeShazer D, Woods DE (1998). *Burkholderia thailandensis* sp. nov., a *Burkholderia pseudomallei*-like species. Int J Syst Bacteriol.

[7] Thibault FM, Hernandez E, Vidal D, Girardet M, Cavallo JD (2004). Antibiotic susceptibility of 65 isolates of *Burkholderia pseudomallei* and *Burkholderia mallei* to 35 antimicrobial agents. J Antimicrob Chemother.

[8] Gilad J (2007). *Burkholderia mallei* and *Burkholderia pseudomallei*: the causative micro-organisms of glanders and melioidosis. Recent Pat Antiinfect Drug Discov.

[9] Schmoock G, Ehricht R, Melzer F, Rassbach A, Scholz HC, Neubauer H (2009). DNA microarray-based detection and identification of *Burkholderia mallei*, *Burkholderia pseudomallei* and Burkholderia spp. Mol Cell Probes.

[10] Scholz HC, Joseph M, Tomaso H, Al Dahouk S, Witte A, Kinne J (2006). Detection of the reemerging agent *Burkholderia mallei* in a recent outbreak of glanders in the United Arab Emirates by a newly developed *fli*P-based polymerase chain reaction assay. Diagn Microbiol Infect Dis.

[11] Tomaso H, Scholz HC, Al Dahouk S, Pitt TL, Treu TM, Neubauer H (2004). Development of 5′ nuclease real-time PCR assays for the rapid identification of the *Burkholderia mallei//Burkholderia pseudomallei* complex. Diagn Mol Pathol.

[12] Lowe W, March JK, Bunnell AJ, O’Neill KL, Robison RA (2013). PCR-based methodologies used to detect and differentiate the *Burkholderia pseudomallei* complex: *B. pseudomallei*, *B. mallei*, and *B. thailandensis*. Curr Issues Mol Biol.

