# Supplementary material for: Clear distinction between Burkholderia mallei and Burkholderia pseudomallei using fluorescent motB primers
Source: Acta Vet Scand. 2015 Mar 7;57(1):13. doi: 10.1186/s13028-015-0104-4 (PMC4364355; doi:10.1186/s13028-015-0104-4)

Additional file 1 Comparison of the *mot*B PCR assay to the conventional *fli*P and real time *fli*C PCR assays in clinical samples (*Burkholderia* type strains ATCC 23343 [T], ATCC 23344[T])

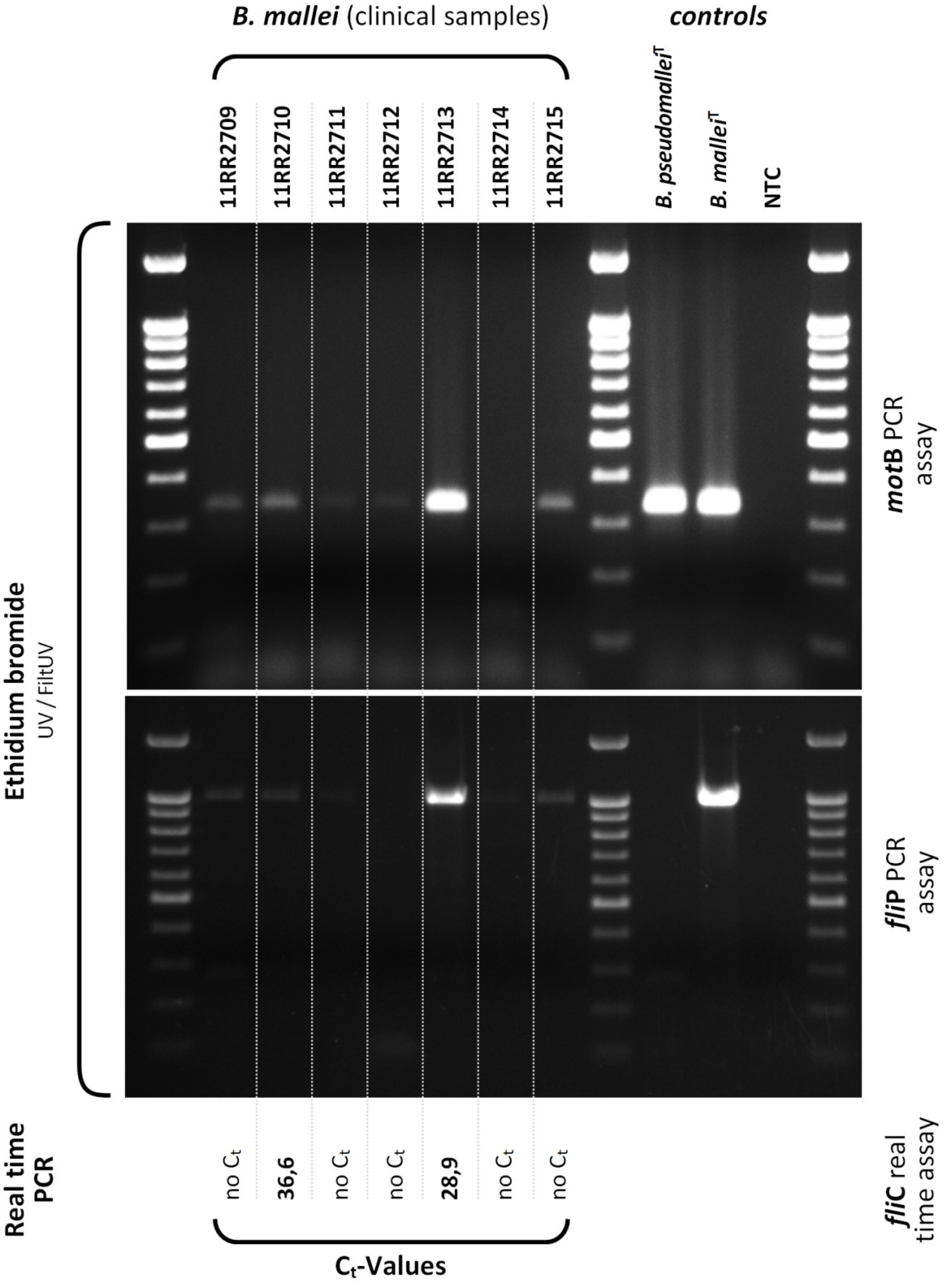

Supplement: Additional file 1: — Comparison of the mot B PCR assay to the conventional fli P and real time fli C PCR assays in clinical samples ( Burkholderia type strains ATCC 23343 T, ATCC 23344T). [file 13028_2015_104_MOESM1_ESM.pdf]
